# Complete genome sequences of *Vibrio coralliilyticus* strains ATCC BAA-450 and OCN014

**DOI:** 10.1128/mra.00069-25

**Published:** 2025-07-21

**Authors:** Victoria N. Lydick, Ram Podicheti, Douglas B. Rusch, Blake Ushijima, Dor Salomon, Julia C. van Kessel

**Affiliations:** 1 Department of Biology, Indiana University 123993, Bloomington, Indiana, USA; 2 Center for Genomics and Bioinformatics, Indiana University 151786, Bloomington, Indiana, USA; 3 Department of Biology and Marine Biology, University of North Carolina Wilmington 14621, Wilmington, North Carolina, USA; 4 Department of Clinical Microbiology and Immunology, School of Medicine, Faculty of Medical and Health Sciences, Tel Aviv University 616193, Tel Aviv, Israel; University of Maryland School of Medicine, Baltimore, Maryland, USA

**Keywords:** *Vibrio*, coral, *Vibrio coralliilyticus*

## Abstract

Here, we announce the complete bacterial genome sequences of *Vibrio coralliilyticus* strains ATCC BAA-450 [3.48 Mb, 1.89 Mb, and pVC450 (381.33 kb)] and OCN014 [3.44 Mb, 1.86 Mb, and pVC14 (397.48 kb)] that comprise two chromosomes and one plasmid, respectively. This effort supports future genomic studies of *V. coralliilyticus* pathogenicity and host species.

## ANNOUNCEMENT

*Vibrio coralliilyticus* is a Gram-negative bacterium and an effective marine pathogen. Records revealed *V. coralliilyticus-*infected hosts including shellfish, finfish, sea urchins, and corals in environments along global coastlines (1) and among United States aquaculture hatcheries (2, 3). Notably, reports indicated host specificity to coral species (2, 4–7). The majority of *V. coralliilyticus* studies demonstrated the causal relationship between inoculation and disease, but recent studies discovered the cellular processes regulating potential virulence factors (1, 8–16).

Strain ATCC BAA-450 (BAA-450), also known as YB1 and LMG 20984, was isolated from a diseased colony of *Pocillopora damicornis* originating from the African archipelago, Zanzibar (6, 17). Strain OCN014 was isolated from a diseased colony of *Acropora cytherea* near the western reefs of Palmyra Atoll in the Pacific Ocean (18). Before this project, *V. coralliilyticus* BAA-450 was partially sequenced under GenBank accession AJ440005 in 2003 (6) and submitted as contigs under GenBank assembly GCA_000176135.1 in 2009 (BioProject: PRJNA40491). OCN014 was partially sequenced and assigned the GenBank accessions CP009264 (Chr1), CP009265 (Chr2), and CP009266 (pOCN014) (BioProject: PRJNA258550) in 2014 (18).

The rationale for this work was to generate new sequence data and complete these two strain genomes to enable genomic and genetic studies of *V. coralliilyticus* coral pathogenesis. *V. coralliilyticus* strains were grown at 30°C on solid glycerol artificial seawater medium and were inoculated into liquid lysogeny broth (LB) marine (LM) medium (LB supplemented to a total of 20 g NaCl L^−1^). Genomic DNA was isolated using the EZ-10 Spin Column Bacterial Genomic DNA miniprep Kit (BioBasic), and the DNA was confirmed via gel electrophoresis and quantified using a Qubit 4. The BAA-450 and OCN014 libraries were sequenced utilizing the Oxford Nanopore Technology (ONT) through Plasmidsaurus. The ONT library was prepared using the Rapid Barcoding Kit 96 V14 (part number SQK-RBK114.96) and a PromethION P24 with R10.4.1 flow cell instrument. Base calling was performed at Plasmidsaurus using the ONT software Guppy version 6.5.7 GPU. Sequenced reads were quality filtered and cleaned using Filtlong v0.2.1 (19) with default parameters and Miniasm v0.3 (20). Reads were downsampled to 100× coverage and assembled using Flye v2.9.1 (21) with parameters selected for high-quality ONT reads (Table 1). The assembly was polished using Medaka v1.8.0 (22) and annotated using Bakta version v1.6.1 (23). The contigs were visualized using Bandage version v0.8.1 (24). The genome completeness was validated and verified for any contamination using CheckM version v1.2.2 (25). The species and plasmid identification were done using Mash version v2.3 (26), Sourmash version v4.6.1 (27), and CheckM version v1.2.2 (25). The assembled contigs were ordered and reoriented by aligning them to the existing GenBank assemblies of *V. coralliilyticus* strains OCN008 (GCA_011212705.1) and OCN014 (GCA_000763535.2) using nucmer from MUMmer version 3.23 (28). The ordered contigs were concatenated with each other and separated by 100 Ns into super contigs. The circularity of the assembled genome was further confirmed by establishing the presence of read sequences spanning the chromosome ends.

**TABLE 1:** Genome assembly data information

	BAA-450	OCN014
Total number of bases sequenced	874,048,408	235,445,132
Total number of reads sequenced	236,643	56,330
Longest read length	65,347	63,549
Median read length	2,445.0	2,738.5
Estimated raw coverage	153×	40×
Assembled coverage	97×	38×
Genome size	5,709,339	5,760,322
N50 for assembly	501,164	1,236,477
rN50^*a*^	5,721	6,505
Number of contigs	5	4
Number of annotated genes	5,302	5,377

^*a*^ rN50 is the “read N50,” which is defined as the shortest read that covers 50% of the total number of bases in the reads used for assembly.

Both *V. coralliilyticus* genomes, BAA-450 and OCN014, were submitted under BioProject PRJNA1105701. The BAA-450 genome (BioSample SAMN41107814; TaxID: 190893) has a G+C content of 45.5% and comprises two chromosomes and a megaplasmid. Chromosome 1 (3,482,485 bp) was assembled from two contigs separated by 100 Ns, using the assembled genome of *V. coralliilyticus* OCN008 (29) (GenBank assembly GCA_011212705.1; NCBI RefSeq assembly GCF_011212705.1) as a scaffold. The same OCN008 scaffold was used to assemble BAA-450 chromosome 2 (1,896,499 bp) and its megaplasmid (coined pVC450) (381,338 bp). Similarly, the OCN014 genome (BioSample SAMN41107815; TaxID: 190893) has a G+C content of 46% and contains two chromosomes and a megaplasmid. Chromosome 1 (3,442,478 bp) was assembled via overlapping forward and reverse complement contigs and compared to the OCN008 genome. The remaining OCN014 genome includes Chromosome 2 (1,869,373 bp) and a megaplasmid (coined pVC14) (397,488 bp).

## Data Availability

From this project, *V. coralliilyticus* BAA-450 and OCN014 were filed under BioProject number PRJNA1105701. The raw read sequences are available under Sequence Read Archive (SRA) accessions SRR32183239 for BAA-450 and SRR32183238 for OCN014. BAA-450 Chr 1, Chr 2, and pVC450 assemblies are submitted under GenBank accessions CP156656, CP156657, and CP156658, respectively. OCN014 Chr 1, Chr 2, and pVC14 are available under GenBank accessions CP156653, CP156654, and CP156655, respectively.
